# Clinical Evaluation of a New Automated Bridge Assay for the Detection of Thyroid-Stimulating Hormone Receptor Autoantibodies

**DOI:** 10.1055/a-2797-6441

**Published:** 2026-02-23

**Authors:** Patricia Schott-Ohly, Samer Albeiruty, Mareike Muchalla, Ursula Doetter, Jann Hommen, Tanja Eberwein, Christiana Vogkou, Derik Hermsen, Joerg Timm, Colin MacKenzie, Frederik Giesel, Matthias Schott

**Affiliations:** 1 537347 Division for Specific Endocrinology, University Hospital Duesseldorf Centre for Health and Society, Duesseldorf, Germany; 2 Division for Specific Endocrinology, Medical Faculty, University Hospital Duesseldorf, Duesseldorf, Germany; 3 537347 Institute of Clinical Chemistry and Laboratory Medicine, University Hospital Duesseldorf Centre for Health and Society, Duesseldorf, Germany; 4 537347 Institute for Virology, University Hospital Duesseldorf Centre for Health and Society, Duesseldorf, Germany; 5 537347 Institute of Medical Microbiology and Hospital Hygiene, University Hospital Duesseldorf Centre for Health and Society, Duesseldorf, Germany; 6 Clinic for Nuclear Medicine, University Hospital Duesseldorf, Duesseldorf, Germany

**Keywords:** TSH receptor antibodies, thyroid, Gravesʼ disease, autoimmune thyroiditis, bridge assay

## Abstract

Graves’ disease is an autoimmune disease caused by autoantibodies to the thyroid-stimulating hormone receptor, which usually leads to hyperthyroidism in these patients. The aim of the present study was to evaluate a new automated bridge chemiluminescence thyroid-stimulating hormone receptor autoantibody assay for the diagnosis and in the follow-up of Graves’ disease patients and to compare this assay with another established competition assay. Altogether, 132 Graves’ disease, 39 autoimmune thyroiditis, 28 non-autoimmune nodular goiter and 27 thyroid cancer patients were included in this study. Receiver-operating characteristic plot analysis based on above mentioned samples revealed an area under the curve of 0.9994 (95% confidence interval: 0.9980–1.001), indicating high sensitivity and specificity of the assay. The optimal sensitivity (99.22%; 95% confidence interval: 95.7–99.8%) and specificity (98.98%; 95% confidence interval: 94.4%–99.8%) were seen at a cut-off level of 0.550 IU/L. The corresponding positive predictive value was 99.22%, whereas the negative predictive value was estimated to be 98.98%. A detailed comparison of the two assay systems used revealed a slightly different thyroid-stimulating hormone receptor autoantibody distribution with the new bridge assay detecting more thyroid-stimulating hormone receptor autoantibody-positive follow-up patients with active Graves’ disease compared with the competition assay. The measurement of thyroid-stimulating hormone receptor autoantibodies revealed a steady decline over the time of follow-up. In summary, our results demonstrate that the new automated bridge assay for detecting thyroid-stimulating hormone receptor autoantibodies has high sensitivity and specificity for diagnosing Graves’ disease and to discriminate from other thyroid diseases, respectively.

## Introduction

Graves’ disease (GD) is an organ-specific autoimmune disease of the thyroid gland that predominantly affects women between 30 and 50 years of age and is the most common cause of hyperthyroidism in iodine-replete populations 1​ with an annual incidence of 20–40 cases per 1,00,000 population. 2​ 3​ ​4​ The mechanism of hyperthyroidism in GD involves the production of autoantibodies to the thyroid stimulating hormone receptor (TSH-R) that mimic the effects of thyrotropin. The TSH-R belongs to the family of 7TM G-protein coupled receptors and is expressed by thyroid follicular cells and, to a much lower level, by thymocytes and fibroblasts of retro-orbital tissue. 5​ Based on their functional effects on the TSH-R, TSH-R antibodies (TRAb) are classified into three types: stimulating, blocking, and cleaving (“neutral” in biological activity terms). 6​ Stimulating TRAb are the most common and the cause of hyperthyroidism in GD patients. 7​ 8​ ​9​

For the quantification of TRAb and confirmation of clinical diagnosis, three different types of assays are commonly used in laboratory medicine. The most widely used assays measure the competition between the binding of TRAb and TSH 10​ ​11​ or a TSHR directed human monoclonal autoantibody, 12​ respectively, at the TSH-R. The latter assay also exists in an automated system. 12​ In contrast to these in vitro TSH competition assays, bioassays measure the increased production of cyclic adenosine monophosphate (AMP) in cellular systems. 13​ 14​ 15​ ​16​ These assays allow us to distinguish stimulating from blocking TRAb. A third assay method for TRAb detection is based on a bridge technology. 17​ Within this assay, autoantibodies are detected by binding with one arm to a capture receptor on the solid phase and bridging with the other arm to a detection receptor giving a signal. The assay uses chimeric TSHRs detecting thyroid stimulating immunoglobulins based on an understanding of the structure of the extra-cellular domain of the TSHR and its interactions with anti-TSHR antibodies. 18​ ​19​ This assay also exists in an automated version. 20​ Importantly, we have shown that this assay does not solely detect stimulating TRAb as claimed by the manufacturer. 21​

Most recently, a new TRAb chemiluminescence immunoassay also using a bridge technology (chemiluminescence immunoassay [CLIA]) has been established (Diasorin). The aim of the present study was to evaluate the performance of the TRAb bridge assay in a clinical setting and to compare it with another conventional automated TRAb competition assay. Altogether, 226 sera of patients with different thyroid diseases have been analyzed.

## Materials and methods

### Patients

Altogether, 226 individuals were included in the study. Of these, 132 suffered from GD (77% woman; mean age: 46 y; and range: 18–88 y), 39 suffered from autoimmune thyroiditis (AIT; 85% woman; mean age: 51 y; and range: 18–74 y), 28 had non-autoimmune nodular thyroid disease (68% woman; mean age: 55 y; and range: 25–80 years) and 27 had thyroid cancer (44% woman; mean age: 58 y; and range: 30–81 y). Of the GD patients, 60 had newly diagnosed disease, whereas 72 GD patients already received antithyroid drugs or ablative therapy had been performed (among them four with inactive disease).

The criteria for GD were based on initially documented hyperthyroidism with or without ophthalmopathy and an increased uptake in technetium scintigraphy or hypoechogenicity and an increased blood flow in ultrasound, respectively. The criteria for hyperthyroidism included clinical symptoms, increased serum concentrations of free T4 and free T3 and decreased basal TSH levels. The criteria for an AIT were the presence of positive anti-thyroperoxidase-autoantibodies (anti-TPO-Ab) and/or anti-thyroglobulin autoantibodies (anti-TG-Ab) without signs of GD and subacute thyroiditis, respectively. This study was approved by the ethical committee of the Medical Faculty of the Heinrich-Heine-University Duesseldorf (No. 2024-2726).

### Thyroid functional tests and antibody assays

The serum concentrations of TSH (reference range: 0.3–4.1 IU/L and lower detection limit: 0.01 lU/l), free T4 (normal range: 9.1–19.1 pg/ml) and free T3 (2.6–5.1 ng/L) as well as anti-TG-Ab (<40 IU/L) and anti-TPO-Ab (<35 IU/L) were measured using commercially available electrochemiluminescence assays from Roche Diagnostics (Cobas System).

### Detection of thyroid-stimulating hormone receptor antibodies

As described by the manufacturer, the method for the determination of TRAb is a bridge CLIA (Diasorin). The method for the determination of TRAb is a sandwich CLIA. The test employs a pair of recombinant hTSH-R constructs (the capture reagent biotinylated human TSHR-Fc and the detection reagent hTSH-R-MBP) in a two step reaction. During the first step, the antibody present in samples or controls forms a bridge between the two receptors and the antigen–antibody–antigen immune complex is then captured by streptavidin-coated magnetic particles (solid phase). The unbound material is removed with a wash cycle. During the second step, a monoclonal antibody anti-MBP labelled with an isoluminol derivative is used as the detection reagent and binds the immune complex previously formed. After this incubation, a second wash cycle removes the unbound material. Subsequently, the starter reagents are added and a flash chemiluminescence reaction is thus induced. The light signal, and hence the amount of the isoluminol–antibody conjugate, is measured using a photomultiplier as relative light units (RLU) and is indicative of the Ab concentration present in calibrators, samples or controls. As described by the manufacturer, the assay is referenced to the WHO second International Standard for TRAb NIBSC 08/204.

This assay was compared with the Elecsys anti-TSH-R electrochemiluminescence immunoassay (ECLIA) measured using a Cobas 8,000 e 801 analyzer (Roche Diagnostics GmbH, Mannheim, Germany). The assay was performed following the manufacturer’s instructions using a cut-off of 1.75 IU/L. As described by the manufacturer, this assay is calibrated against the first IS 90/672 Standard.

### Definition of cut-off and statistical analysis

To obtain the optimal decision threshold level for positivity, receiver-operating characteristic (ROC) analysis was performed. 22​ Sensitivity/specificity pairs were calculated by varying the decision threshold levels over the entire range of TRAb values. The sensitivity (the true positive results) was calculated from patients with GD. The specificity (the true negative results) was calculated from all patients with non-autoimmune nodular thyroid disease, AIT and thyroid cancer patients (excluding GD patients). Four GD follow up patients with inactive disease and confirmed TRAB negative also by the competition assay were included in the true negative results. The positive predictive value (PPV) was calculated as follows: PPV=number of antibody true positive GD patients as a fraction of the total number of antibody positive subjects (true and false positive subjects). The negative predictive value (NPV) was calculated as follows: NPV=number of true antibody negative GD patients as a fraction of the total number of antibody negative subjects (true and false negative subjects). Comparison was done by the analysis of variance test and Dunnett’s multiple comparison test (for data showing a Gaussian distribution) or the Kruskal–Wallis test and the Mann–Whitney test (for not normally distributed data) calculated with Prism computer software (GraphPad Software Inc., San Diego, CA). Correlation analysis was performed with Spearman’s test. A *p* value of less than 0.05 was considered statistically significant.

## Results

### Comparison of proposed and calculated cut-off

In order to independently calculate the sensitivity and specificity of the assay, we first performed a ROC plot analysis. This analysis has been compared with the manufacturer’s recommendations. Altogether, 94 patients with different thyroid diseases were included for calculating specificity and 128 patients with GD were used for calculating sensitivity (four GD patients with long disease duration and with inactive disease were included in specificity evaluation, as expected negative).

**Fig. 1:**
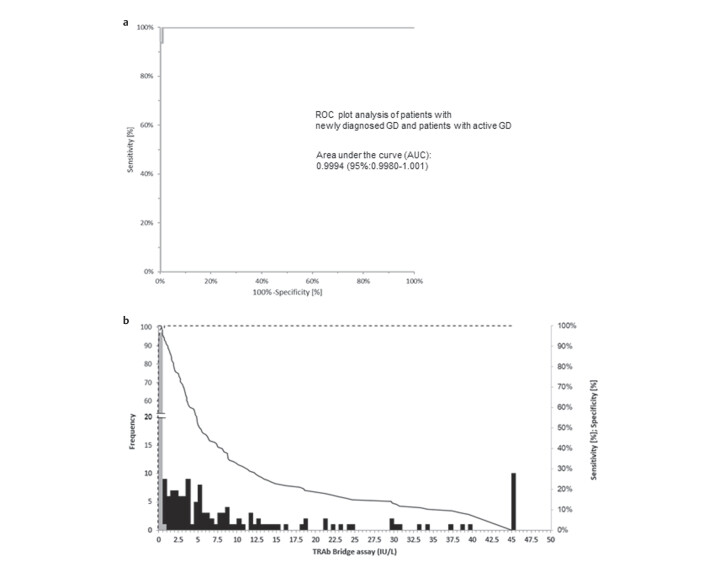
The ROC plot and Gerhardt plot of the new TRAb assay including newly diagnosed GD patients as well as GD patients during the follow-up. ( **a** ) ROC plot analysis including the data of patients with newly diagnosed GD as well as GD patients with active disease for sensitivity and other subjects including patients with AIT, non-autoimmune nodular goiter, thyroid cancer and GD patients within inactive disease for specificity. ( **b** ) The Gerhardt plot showing the frequency distribution of subjects dependent on the TRAb serum level. Grey bars represent expected negative samples. Black bars represent Graves’ expected positive disease patients. Based on these data, the calculated sensitivity (solid line) and specificity (dashed line) are shown. TRAb values of>40 IU/L were estimated as ‘45’. AIT, autoimmune thyroiditis; GD, Graves’ disease; ROC, receiver-operating characteristic; TRAb, thyroid-stimulating hormone receptor autoantibodies.

The area under the curve (AUC) for the automated bridge assay was 0.9994 (95% confidence interval [CI]: 0.998–1.001; Fig. 1a ). The optimal sensitivity (99.22%; 95% CI: 95.7–99.8%) and specificity (98.98%; 95% CI: 94.4–99.8%) were seen at a cut-off level of 0.550 IU/L, proposed by the manufacturer. The corresponding PPV was 99.22%, whereas the NPV was estimated to be 99.98%. The sum of these data was also calculated and illustrated within a Gerhardt plot ( Fig. 1b ). The sensitivity of the competition assay was 94.53% (95% CI: 89.1–97.3%), whereas the specificity was 100% (95% CI: 96.2–100%; Fig. 1b ).

In addition, we also calculated the sensitivities and specificities of both assays using only newly diagnosed GD patients. Here, the sensitivity of the bridge assay at a cut-off level of≥0.550 IU/L was 98.33% (95% CI: 91.1–97.7%) and the specificity was 98.94% (95% CI: 94.2–99.8%). The corresponding PPV was 98.33%, whereas the NPV was estimated to be 98.94%. For the competition assay, the sensitivity was 98.33% (95% CI: 91.1–97.7%) and the specificity was 100% (95% CI: 96.1–100%).

### Clinical evaluation of the new bridge assay and comparison with the automated competition assay

**Fig. 2:**
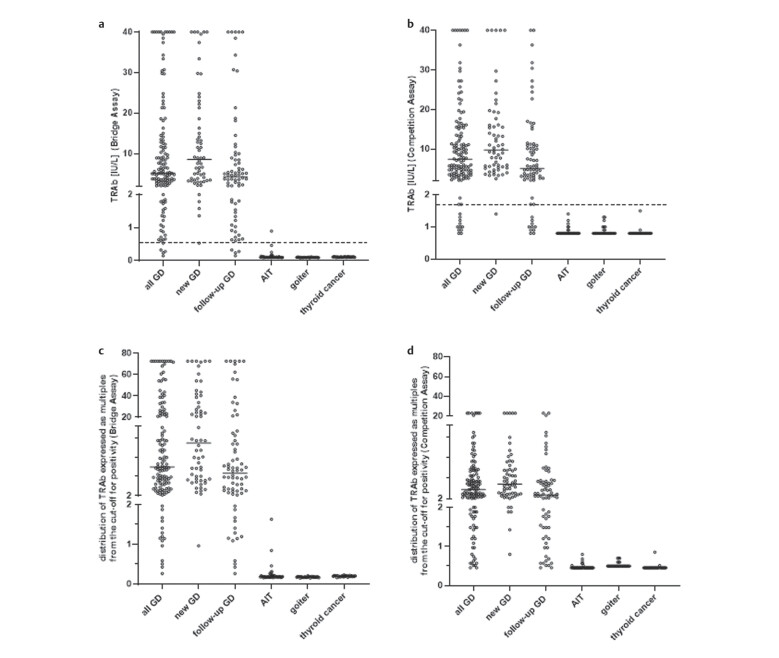
TRAb levels as well as magnitudes of the cut-off for positivity. Distribution of measured TRAb levels (( **a** ) bridge assay and ( **b** ) competition assay) and distribution of TRAb expressed in multiples of the cut-off for positivity (( **c** ) bridge assay and ( **d** ) competition assay) in patients with GD (all GD) independently of the disease stage, new-onset GD (new GD), GD during the follow-up, autoimmune thyroiditis (AIT), non-autoimmune goiter patients (goiter), and thyroid cancer. As based on our analyses or recommended by the manufacturers, respectively, the following cut-offs for TRAb positivity have been used: bridge assay for the detection of TRAb: 0.550 IU/L and competition assay for the detection of TRAb: 1.75 IU/L. For all GD, the distribution of TRAb multiples measured by the bridge assay differs significantly from TRAb multiples of the cut-off of the competition assay: by using a cut-off of “2-times” positivity the relationship of positive to negative patients was significantly higher within the bridge assay compared to the competition assay (117/15 patients vs. 100/32 patients; *p* <00001). GD, Graves’ disease; TRAb, thyroid-stimulating hormone receptor autoantibodies.

In order to evaluate the new assay in routine clinical practice, we measured TRAb not only in patients with GD but also in other thyroid diseases including AIT, non-autoimmune goiter, and thyroid cancer. Both assays significantly detected GD patients ( *p* <0.0001). The automated bridge assay detected a slightly higher percentage within the group of all GD patients with active disease compared to the competition assay (127/128; 99.22% vs. 121/128; 94.53%; *p* =0,025). By including the four GD patients with inactive disease, the number of TRAb positive patients was 127/132 (96,21%) for the bridge assay and 121/132 (91,67%) for the competition assay ( Fig. 2a, b ). Within the group of GD patients with newly diagnosed disease, the new bridge assay detected the same number of patients (59/60; 98.33%) compared to the competition assay.

By analyzing non-GD patients, differences were seen in the group of AIT patients: by using the new bridge assay, TRAb above the cut-off for positivity was seen in 1 of 39 AIT patients (2.56%), whereas in the competition assay no AIT patient was detected. In the group of goiter patients as well as thyroid cancer patients, no TRAb were detected in both assays tested.

Because of different thresholds for TRAb positivity which is caused by calibration against two different WHO standards, thresholds (cut-offs) for positivity were estimated as ‘1’ and differences were expressed as multiples from this cut-off ( Fig. 2c, d ). Again, a similar pattern was seen for both assays. A more detailed analysis, however, revealed significant pattern differences by comparing both assays ( Fig. 2c, d ). By using a cut-off of “2-times” positivity, the relationship of positive to negative patients was significantly higher within the bridge assay compared to the competition assay (117/15 patients vs. 100/32 patients; *p* <0.0001).

**Fig. 3:**
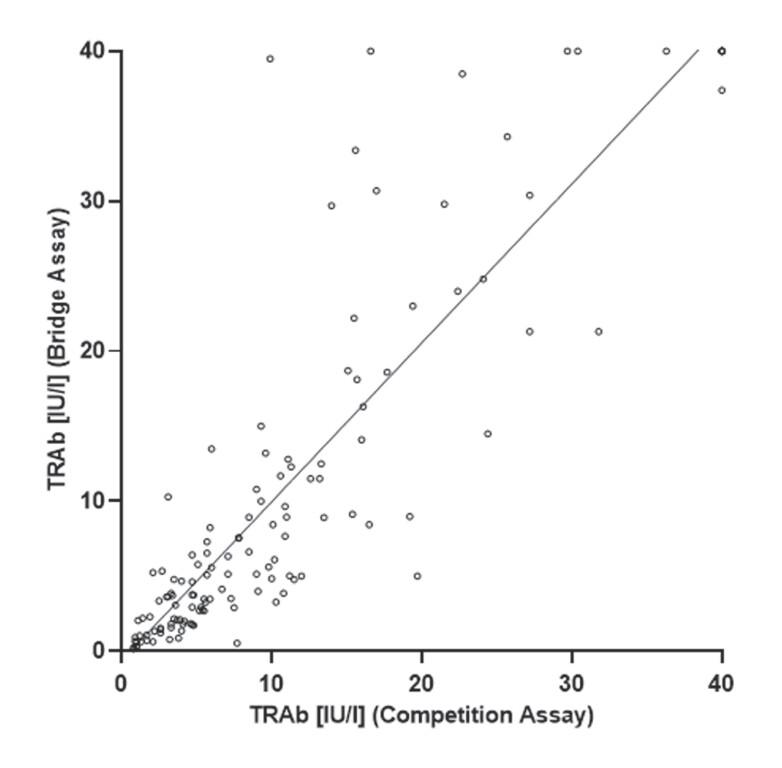
Correlation between TRAb assays. A significant correlation of TRAb levels using both assay systems for all GD patients was seen (Spearman’s *r* =0.8795; *p* <0.0001). GD, Graves’ disease; TRAb, thyroid-stimulating hormone receptor autoantibodies.

Additionally, to compare both assay systems, a correlation analysis of the new bridge assay and the competition assay was done. A strong correlation between both assays was seen (Spearmanʼs *r* =0.8795; *p* <0.0001, Fig. 3 ).

### Thyroid-stimulating hormone receptor autoantibodies during follow-up

**Fig. 4:**
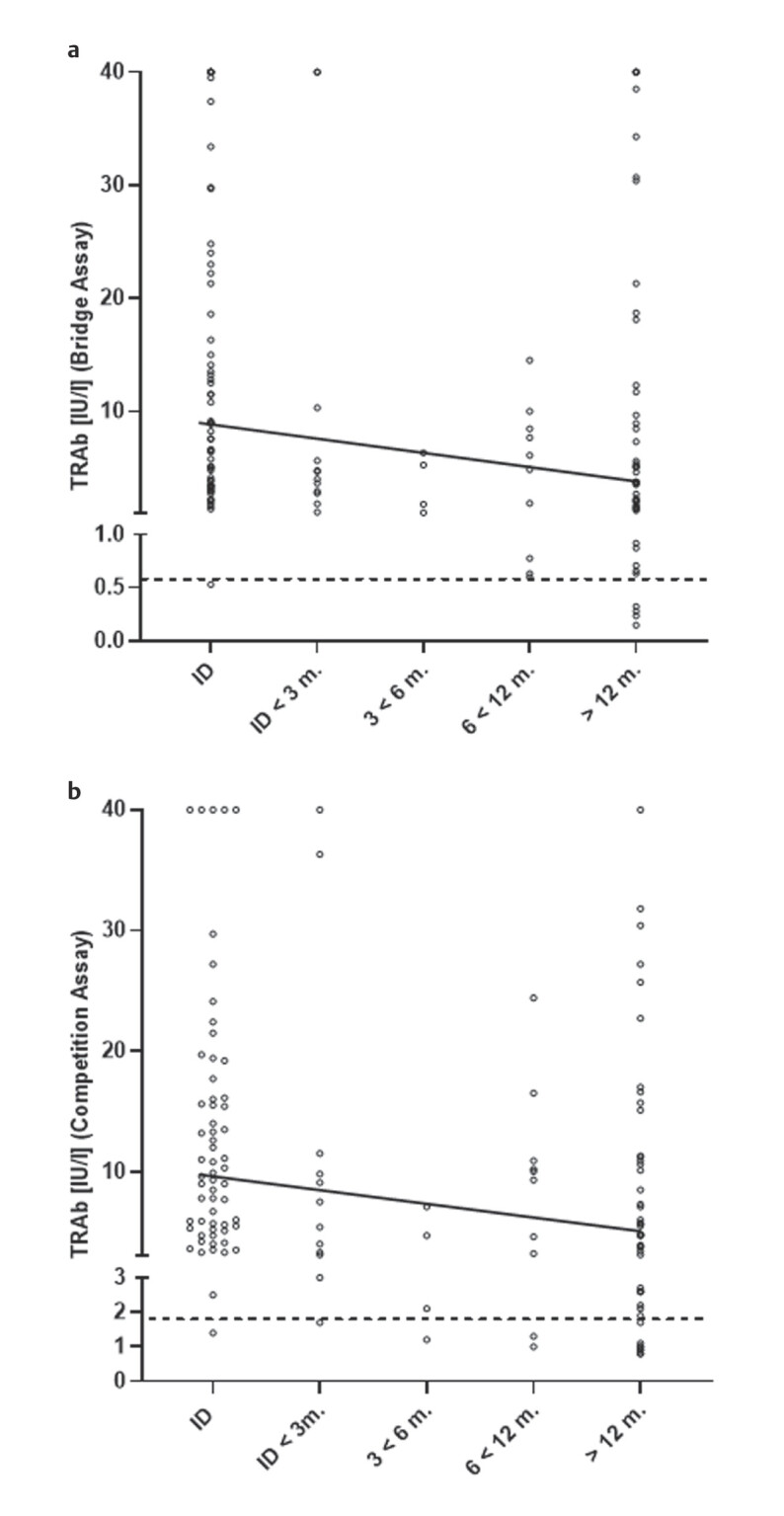
TRAb measured in both assay systems in dependency to the time point of the initial diagnosis (ID) of GD and during follow-up. TRAb (( **a** ) bridge assay system) and TRAb (( **b** ) competition assay system) levels in dependency to the time since the ID of GD are shown. The longer the interval between the ID and the measurement, the lower is the level of TRAb in both assay systems (bridge assay: Spearman’s *r* =− 0.004546; *p* =ns; competition assay: Spearman’s *r* =− 0.08818; *p* =ns). GD, Graves’ disease; TRAb, thyroid-stimulating hormone receptor autoantibodies.

As shown in Fig. 4a , there was a clear decline of TRAb during follow-up of more than 12 months after initial diagnosis in the bridge assay (Spearman’s *r* =−0.004546, *p* =ns). A similar picture was seen in the same group of patients by testing for TRAb in the competition assay (Spearman’s *r* =−0.08818, *p* =ns; Fig. 4b ).

### Correlation between thyroid-stimulating hormone receptor autoantibodies and free thyroxine

**Fig. 5:**
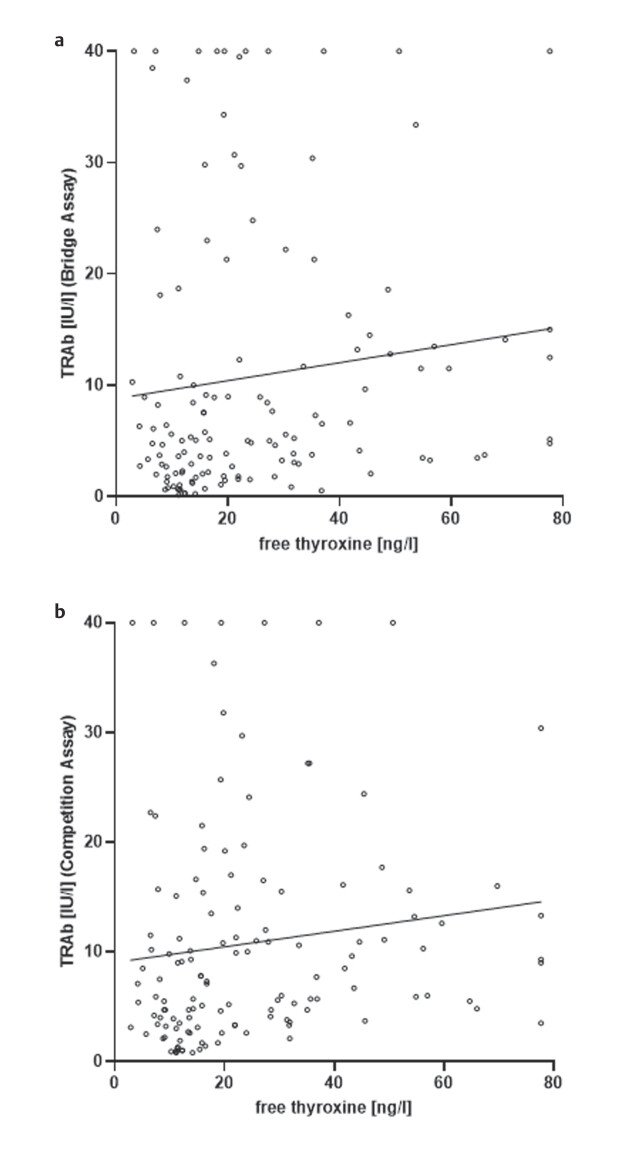
Correlation between TRAb assays and fT4. Correlation of TRAb levels (( **a** ) bridge assay system and ( **b** ) competition assay system) to free thyroxine (fT4) of all GD patients. Both, TRAb measured in the bridge as well as in the competition assay system correlate to the fT4 level (bridge assay: Spearman’s *r* =0.2510, *p* <0.0037; competition assay: Spearman’s *r* =0.2778, *p* <0.0013). fT4, free thyroxine; GD, Graves’ disease; TRAb, thyroid-stimulating hormone receptor autoantibodies.

TRAb values of all GD patients correlated significantly to fT4 values in both assay systems (bridge assay: Spearman’s *r* =0.2510, *p* <0.0037; competition assay: Spearman’s *r* =0.2778, *p* <0.0013; Fig. 5a, b ).

## Discussion

The aim of the present study was to evaluate the new automated bridge assay for the detection of TRAb in patients with GD and to compare these data with those of other thyroid diseases. Additionally, we compared this assay with a conventional automated TRAb assay (competition assay). Based on a ROC plot analysis, the proposed cut-off for positivity for the bridge assay of≥0.550 IU/L results in a sensitivity of 99.22% and a specificity of 98.98%. Our results demonstrate that this new assay system for the detection of TRAb has a high sensitivity for detecting GD and specificity for discriminating GD from other thyroid diseases. We also investigated TRAb levels during the follow-up of the disease. As expected, we found a decline of these antibodies over the time period of follow-up.

We obtained a threshold for TRAb positivity of ≥0.550 IU/L for diagnosing GD patients. This is in line with another bridge TRAb assay, in which we also identified the exact same cut-off for TRAb positivity of≥0.550 IU/L. 20​ Both assays are calibrated against the same second WHO standard. These data indicate that TRAb bridge assays using the same technology and TSH receptor epitopes and which are calibrated against the same WHO standard may have the same cut-off for TRAb positivity at around 0.550 IU/L.

In our study, we noted a slightly higher proportion of AIT patients with borderline TRAb (below the cut-off for positivity) as well as one AIT patient which had reproducible positive TRAb within the bridge assay (data not shown; TRAb 0.89/0.88/0.99 IU/L). Within the competition assay, TRAb were negative even though signals have been detected (TRAb 1.0/1.2 IU/L). Vice versa, one patient with classical AIT for many years which transformed into GD (and which was therefore be classified as newly diagnosed GD) had reproducible positive TRAb on the basis of the bridge assay (data not shown; TRAb 2.20/2.20/2.07 IU/L), whereas no TRAb were detected in the competition assay (TRAb 1.4/1.1/0.8 IU/L). In the past, we and others already reported on the existence of TRAb in AIT patients (without defining their potential stimulating, blocking or neutral activity). Formerly, Kahaly et al. also reported on stimulating TRAb in AIT patients with associated orbitopathy. 23​ Within this study, stimulating TRAb were measured using a manual bioassay described before. The slightly increased prevalence of TRAb measured in the bridge assay compared to TRAb measured in the competition assay is most likely due to the higher analytical performance of the new bridge assay for the detection of TRAb compared to the conventional TBII assay. Our present study confirms this observation. Based on these data, it needs to be discussed whether TRAb positivity in such patients may indicate a yet undefined “mixed” immune response with features of both AIT and GD.

Another important issue is the non-identical distribution of TRAb in both assays. Since both assays tested are calibrated against two different WHO standards, thresholds (cut-offs) for positivity were estimated as ‘1’ and differences of both assays were expressed as multiples from the cut-off ( Fig. 2c, d ). Within the competition assay, all AIT, goiter, and thyroid cancer patients were TRAb negative. On the basis of the bridge assay, only one AIT patient and none of the goiter or thyroid cancer patients were TRAb positive. This indicates a high specificity of the bridge assay for detecting TRAb. These results are also in line with previous published data describing TRAb in AIT patients. 20​

To date, only manual bioassays for the detection of TRAb have been available. 14​ 15​ 16​ ​17​ The drawback of all manual assay systems based on cyclic AMP measurements in cellular systems and independently of their individual sensitivity and specificity is, however, their labour-intensive and time-consuming assay procedure taking several hours. Previously, another bridge assay claimed to only detect stimulating TRAb. 17​ This assay also exists in an automated version. 20​ Importantly, we have shown that this assay does not solely detect stimulating TRAb as claimed by the manufacturer. 21​

This study also has some limitations: (1) our study is a single center analysis and, therefore, this may limit the generalizability of the results. Since our patients are well characterized, this should, however, not affect our findings. (2) We do not have the detailed information of all GD patients regarding the presence of an endocrine ophthalmopathy. Therefore, we did not include these information. It would be very informative, whether EO patients would have higher TRAb titers, which we would expect. (3) Our longitudinal data show that only some GD patients become TRAb negative. This raises the possibility that our cohort represents a subset of more severely affected or difficult-to-treat GD patients. Such a selection bias could influence both the apparent persistence of TRAb positivity and the perceived performance of the assay during the long-term follow-up. Patients with milder disease or earlier remission are certainly under-represented in the long-term follow-up group.

In summary, our results demonstrate the new automated bridge assay to detect TRAb with high sensitivity (in diagnosing GD) and specificity (in discriminating it from other thyroid diseases). Based on our data, the proposed cut-off for TRAb positivity of≥0.550 IU/L is considered adequate. Whether this assay will also help for an improved outcome prediction in GD patients as well as in Graves’ ophthalmopathy needs to be investigated in future studies.
